# Editorial: Surgery and COVID-19: Which Strategies to Apply in Oncologic Patients

**DOI:** 10.3389/fsurg.2021.718751

**Published:** 2021-07-22

**Authors:** Ugo Cioffi, Michele M. Ciulla, Matilde De Simone, Marco Scarci, Alberto Testori, Federico Raveglia, Marco Chiarelli

**Affiliations:** ^1^Department of Surgery, University of Milan, Milan, Italy; ^2^Laboratory of Clinical Informatics and Cardiovascular Imaging, Department of Clinical Sciences and Community Health, University of Milan, Milan, Italy; ^3^Thoracic Surgery, San Gerardo Hospital, Monza, Italy; ^4^Department of General and Thoracic Surgery, Humanitas Research Hospital, Rozzano, Italy; ^5^Emergency and Robotic Surgery, A. Manzoni Hospital, ASST Lecco, Lecco, Italy

**Keywords:** COVID-19, oncologic patients, surgery, viral infection, pandemia

Historically, humans as a species have been cyclically affected by pandemics that, mostly, derive from other species as a consequence of ecosystem breakdowns (1). More recently, the emergence of COVID-19 pandemic, due to the high transmission rate of SARS-CoV-2, overwhelmed the world health systems that faced two problems: the availability of hospital beds, especially ICU beds, and by the number of health personnel contracting the infection, thus, surgical activity has been considerably reduced to give beds and whole wards to COVID-19 patients (2).

In this topic we collected contributions on the strategies to offer the best possible treatment to cancer patients during COVID-19 pandemic, in particular, to set-up and manage COVID-19 free hub for cancer surgery or how to operate on cancer patients, who do not need intensive therapy, evaluating alternative equally effective solutions, with oncologists and radiotherapists, and finally on how to operate on COVID-19 infected patients.

The management of oncologic patients poses unique challenges during the COVID-19 pandemic. Due to the immunosuppressive effects of the malignancy itself and the oncologic treatments (chemotherapy, radiotherapy, and surgery), cancer patients are more susceptible and vulnerable to the COVID-19 infection than the rest of the population (3). Indeed, patients with underlying malignancies, have more severe COVID-19 disease, with an estimated mortality rate of 20–40% (4). In this perspective, Hospitals Departments must be reorganized to ensure the strict division between COVID-19 and COVID-free patients. Moreover, the majority of the Hospitals were forced to reorganize the surgical paths of cancer patients in light of the unavailability of ICU unit beds. Consequently, in the COVID-19 pandemic era *ad hoc* recommendations have been issued by International Scientific Societies to adjust the clinical practice in surgical oncology. Operative indications were reduced and less aggressive therapies were recommended. In this setting, multidisciplinary teams were tasked with verifying, not only the surgical indications but also the treatment priorities.

Clinically the COVD-19 infection poses new diagnostic and therapeutic challenges: in confirmation of this, the article by Baisi et al. illustrates how the virus can cause mediastinal masses simulating lymphomas.

In their review Ciriaco et al. analyzed the worldwide experiences of General Thoracic Surgery Departments in dealing with the diagnostic and therapeutic path of patients with non-small cell lung cancer (NSCLC) during COVID-19 pandemic.

Postoperative mortality after thoracic surgery in patients with COVID-19 is significantly higher than the standard reported mortality. In a COVID-19 positive patient, surgery should be delayed for 2 to 3 weeks unless it is an emergency. In this case, the health care workers must carry out the intervention in a dedicated COVID-room and with every necessary precaution. During the pandemic in Lombardy, surgery was restricted to patients likely to have survival compromised if the operation was not performed within the next weeks. In detail, patients with stage IC, II, III NSCLC or patients who already received induction therapy, surgery was guaranteed in 2–3 weeks. On the other hand, patients with nodules <2 cm in stage I or indolent malignancies were postponed for 1–2 months. The San Raffaele Thoracic Surgery Department in Milano (Italy) suffered a 50% reduction in beds at the two peaks of the pandemic. However, the number of surgeries for patients with NSCLC, compared to the same periods of the previous year, has not undergone substantial changes: only 4% of patients requiring major resection were diverted at the COVID-free hub Hospitals.

In their article Wang et al. from the New York University Langone Health, described the management of NSCLC during pandemic in a Metropolitan Hospital. Their analysis starts from the difficulty to have dedicated non-COVID Centers for oncologic surgery in this emergency condition. Therefore, the Authors describe very accurately the correct paths for treating lung cancer patients during the pandemic. Management should be different based on whether the Hospital system was in surge phase of COVID-19 outbreak or the capacity of the Hospital was saturated. If the City and Hospital are not in a surge phase and had available resources, standard criteria for NSCLC work-up and management are followed with several precautions to avoid the spread of the virus. In limited hospital capacity, all patients have to be discussed with a multidisciplinary approach. The decisions regarding surgical resection should be determined by the growth rate as F-deoxyglucose avidity of the cancer, patient clinical factors and the co-morbidities, including COVID-19 status. Surgery of NSCLC is restricted to patients whose survivorship was likely to be compromised by a surgical delay of 3 months. All procedures essential for cancer staging should be performed and indications for early surgery include solid or predominantly solid (>50%) lung cancer >2 cm, node positive lung cancer and post-induction therapy cancers.

In their manuscript Blache et al. describe the gynecologic surgical activity at Institut Paoli-Calmettes—University of Marseille (France) a COVID free Cancer Center. The Author's priority was to maintain the more urgent and more complex therapeutic procedures during the COVID-19 pandemic, having a “care as usual” strategy during this outbreak. A dedicated COVID unit was created that evaluated all suspected patients and transferred them directly to the COVID units in dedicated Hospitals. The objective of the study was to evaluate the impact of the COVID-19 pandemic on gynecological cancer surgery comparing the surgical activity before and during the national lockdown: their results showed a 33% decrease in activity between the two periods. This reduction mainly concerned the simple diagnostic surgeries. There were no differences between the surgical approaches, the surgical complexity, the length of stay, and the perioperative complication rates. The Authors report a significant decrease in the time of return to intended oncological treatment (RIOT—the time interval between the first day of surgical hospitalization and the return to the intended medical treatment) during the lockdown period. Nevertheless, the implementation of ERAS programs participates in reducing the delay between surgery and adjuvant treatment with a potentially positive impact on the oncologic outcomes. During the COVID-19 pandemic, “care as usual” could represent an acceptable surgical strategy without impairing the oncologic outcome in a Comprehensive Cancer Center with a patient centered clinical pathway for gynecologic cancers.

Pellini et al. describes their approach to breast cancer management during pandemic by “lean thinking.” Lean thinking allows for better performance, increasing both the efficiency and quality of services, while reducing bottlenecks, imperfections, and lead times.

The aim of their study was to compare, breast cancer patients' workflow times at their Breast Unit prior to the outbreak of the COVID-19 pandemic with the workflow times during the COVID-19 pandemic after the implementation of some key interventions. Indeed, the healthcare emergency became an opportunity for the application of new technologies (e.g., indocyanine green fluorescence guided sentinel lymph node biopsy and cryoablation of tumor lumps) and implementation of oncologic therapy (neoadjuvant endocrine therapy) in clinical practice to improve the patient's management flow, despite the limited resources. Through the implementation of these key interventions, the team of Verona University Hospital (Italy) obtained results in breast cancer patients similar to the pre-COVID era, minimizing healthcare personnel and patients' unnecessary risk of SARS-CoV-2 exposure, promoting a rational use of available resources, while at the same time complying with general oncological principles.

The surgical management of cancer patients described by these Authors could offer very interesting models for the optimization of preoperative and postoperative strategies, with a significant impact on patients' perceived quality of care, though amid a global healthcare emergency.

## Author Contributions

All authors listed have made a substantial, direct and intellectual contribution to the work, and approved it for publication.

## Conflict of Interest

The authors declare that the research was conducted in the absence of any commercial or financial relationships that could be construed as a potential conflict of interest.
